# Supplementary material for: Noise-assisted variational quantum thermalization
Source: Sci Rep. 2022 Mar 9;12:3862. doi: 10.1038/s41598-022-07296-z (PMC8907242; doi:10.1038/s41598-022-07296-z)
Supplement: Supplementary file 1 — Supplementary Information. [file 41598_2022_7296_MOESM1_ESM.pdf]

Noise-Assisted Variational Quantum Thermalization

Jonathan Foldager[1*], Arthur Pesah[2], and Lars Kai Hansen[1]

[1]Technical University of Denmark, Department for Applied Mathematics and Computer Science, Kongens Lyngby, 2800, Denmark
[2]University College London, Department of Physics and Astronomy, London WC1E 6BT, United Kingdom
[*]jonf@dtu.dk

SUPPLEMENTARY INFORMATION

Supplementary Note: Estimating the free energy of a noisy circuit

In order to learn the thermal state with NAVQT, we need to minimize the free energy. Obtaining the free energy from the output of a quantum circuit is hard, since the entropy is a highly non-linear function of the state. For unitary evolutions, the entropy is constant, but for non-unitary circuits, including our ansatz, the entropy needs to be estimated for each change of parameters. To simplify this task, we consider the following approximation, represented in Fig. 1c of the main manuscript: all the depolarizing gates are shifted to the beginning of the circuit. Using this circuit, it is now possible to compute the entropy analytically.

If m is the number of layers of our unitary ansatz, the approximate circuit consists in the composition of m depolarizing gates $\mathscr{D}(\lambda)$ for each qubit. We can now use the fact that the composition of depolarizing gates is itself a depolarizing gate:

$$\mathscr{D}(\lambda_2)\circ\mathscr{D}(\lambda_1)=\mathscr{D}(1-(1-\lambda_2)(1-\lambda_1)) \tag{S1}$$

or more generally

$$\mathscr{D}(\lambda_m)\circ...\circ\mathscr{D}(\lambda_1)=\mathscr{D}(1-(1-\lambda_m)...(1-\lambda_1)) \tag{S2}$$

Assuming that the noise parameter λ is the same for all the gates, then the above simplifies to $\mathscr{D}(1-(1-\lambda)^m)$. If we note $\Lambda=1-(1-\lambda)^m$ this new parameter, the entropy of m consecutive noise gates acting on a single qubit initialized with $|0\rangle$ can be written as

$$\begin{aligned}S(\rho_\Lambda)&=-\operatorname{Tr}[\rho_\Lambda\ln(\rho_\Lambda)]\\&=-\operatorname{Tr}\left[\left((1-\Lambda)\,|0\rangle\langle 0|+\Lambda\frac{\mathbb{1}}{d}\right)\ln\left((1-\Lambda)\,|0\rangle\langle 0|+\Lambda\frac{\mathbb{1}}{d}\right)\right]\\&=-\left[\left((1-\Lambda)+\frac{\Lambda}{d}\right)\ln\left((1-\Lambda)+\frac{\Lambda}{d}\right)+\frac{(d-1)\Lambda}{d}\ln\left(\frac{\Lambda}{d}\right)\right]\end{aligned} \tag{S3}$$

and substituting for $\Lambda=1-(1-\lambda)^m$

$$\begin{aligned}S(\rho_\lambda)=-\Bigg[&\left((1-\lambda)^m+\frac{(1-(1-\lambda)^m)}{d}\right)\ln\left((1-\lambda)^m+\frac{(1-(1-\lambda)^m)}{d}\right)\\&+\frac{(d-1)(1-(1-\lambda)^m)}{d}\ln\left(\frac{(1-(1-\lambda)^m)}{d}\right)\Bigg]\end{aligned} \tag{S4}$$

We now use the fact the entropy of a product state is the sum of the individual entropies to write

$$S(\rho_\lambda^{\otimes N})=NS(\rho_\lambda) \tag{S5}$$

which directly gives us the entropy of the state preceding the unitary ansatz. Since applying a unitary operation to a state does not change its entropy, it means that the overall entropy of the output state, that we call $S(\lambda)$, is given by the expression above. To optimize over it, we need to compute its gradient, which can also be obtained analytically as

$$\nabla_\lambda S(\lambda)=N\frac{d-1}{d}m(1-\lambda)^{m-1}\left[-\ln\left(\frac{(1-(1-\lambda)^m)}{d}\right)+\ln\left(\frac{(1-(1-\lambda)^m+d(1-\lambda)^m)}{d}\right)\right] \tag{S6}$$

The overall free energy, which we want to minimize, is given by

$$F(\boldsymbol{\theta},\lambda) = E(\boldsymbol{\theta},\lambda) - TS(\lambda). \tag{S7}$$

The gradient of the energy with respect to θ can be efficiently computed on a quantum device using the parameter shift-rule, while its gradient with respect to λ can be computed using finite-difference (since $E(\lambda)$ itself can easily be extracted from the output of the circuit). Therefore, the overall gradient, given by

$$\nabla_{\boldsymbol{\theta}} F(\boldsymbol{\theta},\lambda) = \nabla_{\boldsymbol{\theta}} E(\boldsymbol{\theta},\lambda) \tag{S8}$$

$$\nabla_{\lambda} F(\boldsymbol{\theta},\lambda) = \nabla_{\lambda} E(\boldsymbol{\theta},\lambda) - T\nabla_{\lambda} S(\lambda) \tag{S9}$$

can be efficiently computed using our circuit approximation.

Supplementary Figures

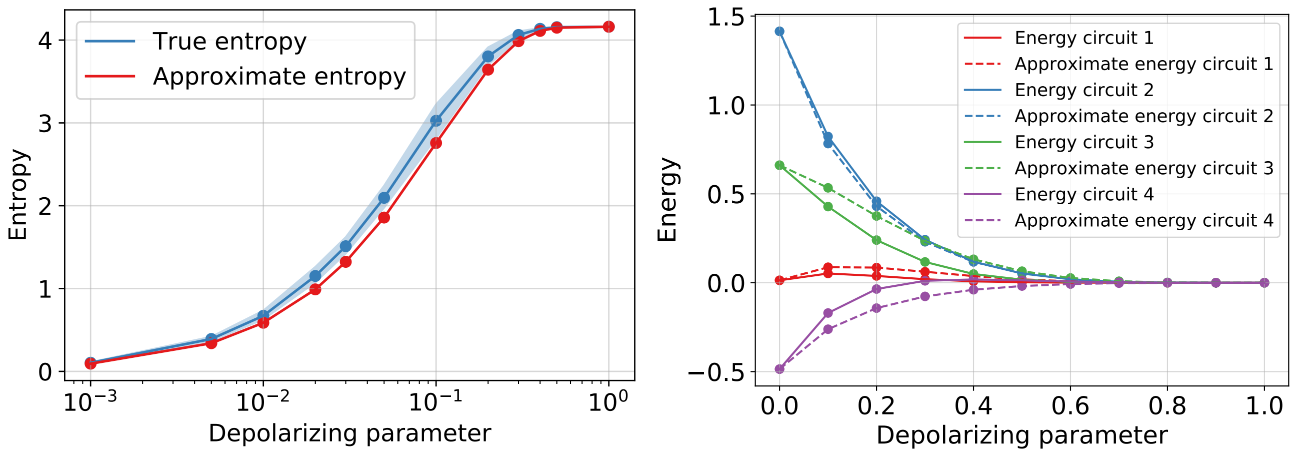

(a) Entropy comparison

(b) Energy comparison

Figure S1. Comparison of the approximate ansatz with the true one, for both the entropy and the energy as a function of the depolarizing noise, for random circuits with 6 qubits and 3 layers. **(a)** Entropy of the two circuit types. Since the entropy of the true circuit depends on the unitary parameters, we sampled 100 random parameters and took the average, minimum and maximum of the entropy (blue area). We see that the two curves follow a similar trajectory, with the approximate entropy being a lower bound on the true one. **(b)** Energy of the two circuit types for the transverse-field Ising model with uniform coefficients. Each color represents a circuit with different random unitary parameters. We see that the approximate energy tends to be close to the true one, following an overall similar trajectory.

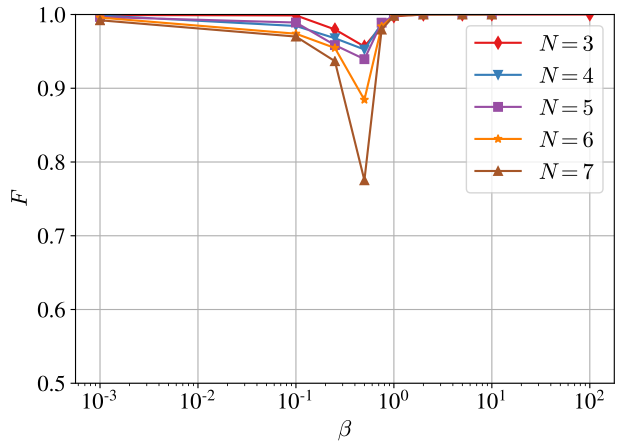

(a) IC with uniform coefficients.

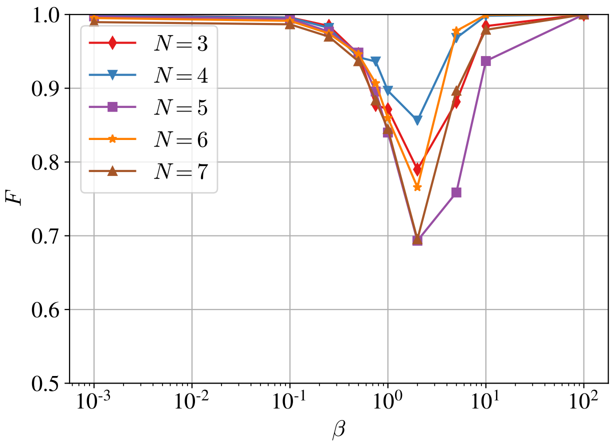

(b) IC with random coefficients.

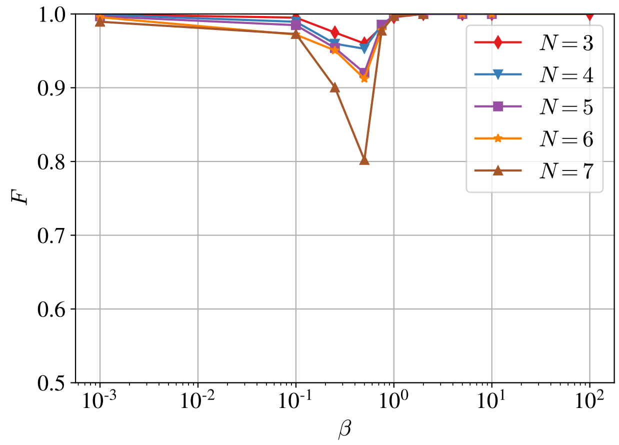

(c) TFI with uniform coefficients.

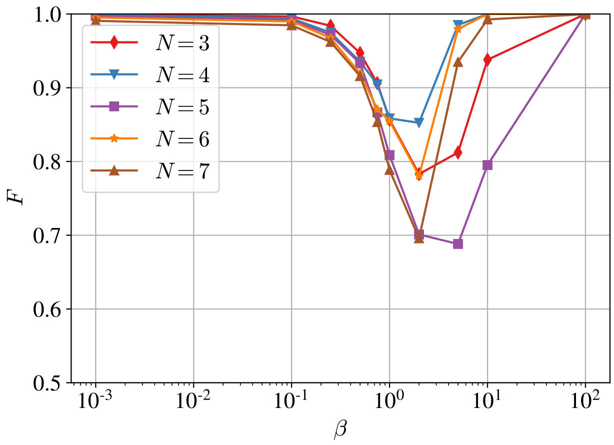

(d) TFI with random coefficients.

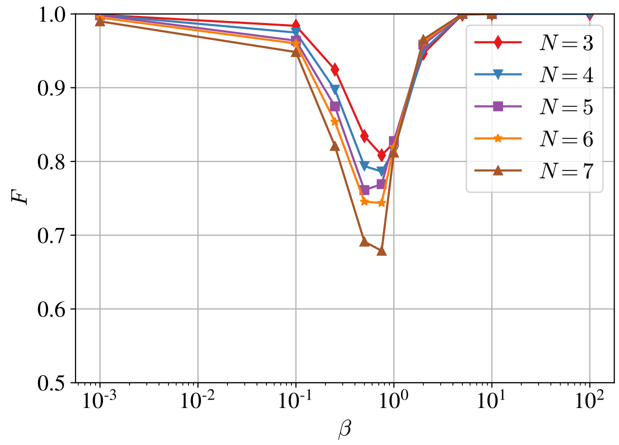

(e) Heisenberg with uniform coefficients.

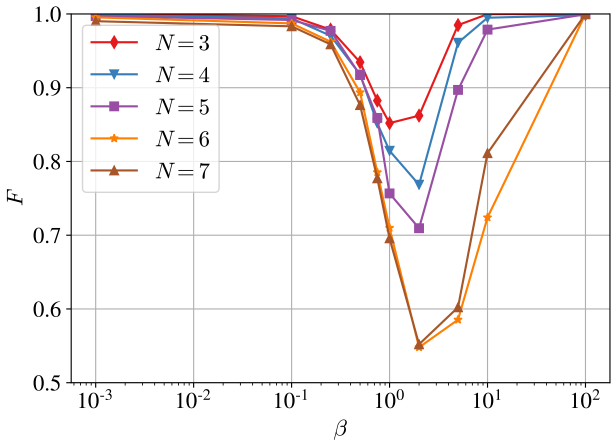

(f) Heisenberg with random coefficients.

Figure S2. Fidelities obtained when minimizing the actual free energy (as opposed to the approximation), using finite-difference to obtain the gradient. We see that the results are similar to those in Fig. 3 of the main manuscript, where the approximate free energy is used for optimization. It shows that the approximation serves as a good heuristics for optimizing our NAVQT ansatz.

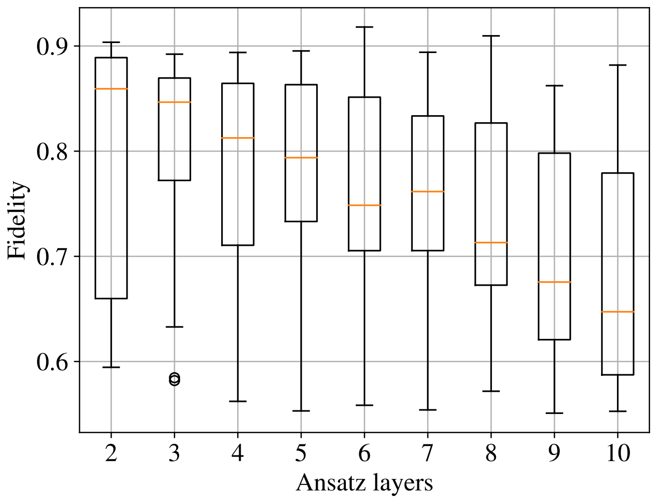

Figure S3. Thermal state fidelity as a function of ansatz layers for 3 qubits Heisenberg model with random coefficients over 5 random seeds. It illustrates that fidelity does not increase with ansatz layers, which suggest that it is not lack of circuit expressivity, but instead either lack of precision in the the free energy approximation and/or that the depolarization channel is not enough for thermalization. A similar pattern to this figure was kept for all the systems we studied. We were able to improve the fidelity slightly when comparing to Fig. 4f ($N = 3$), but we also see that the median fidelity decreases as a function of layers, which we hypothesize this is due to the entropy approximation gets worse with the number of layers and/or that the depolarization noise accumulates.

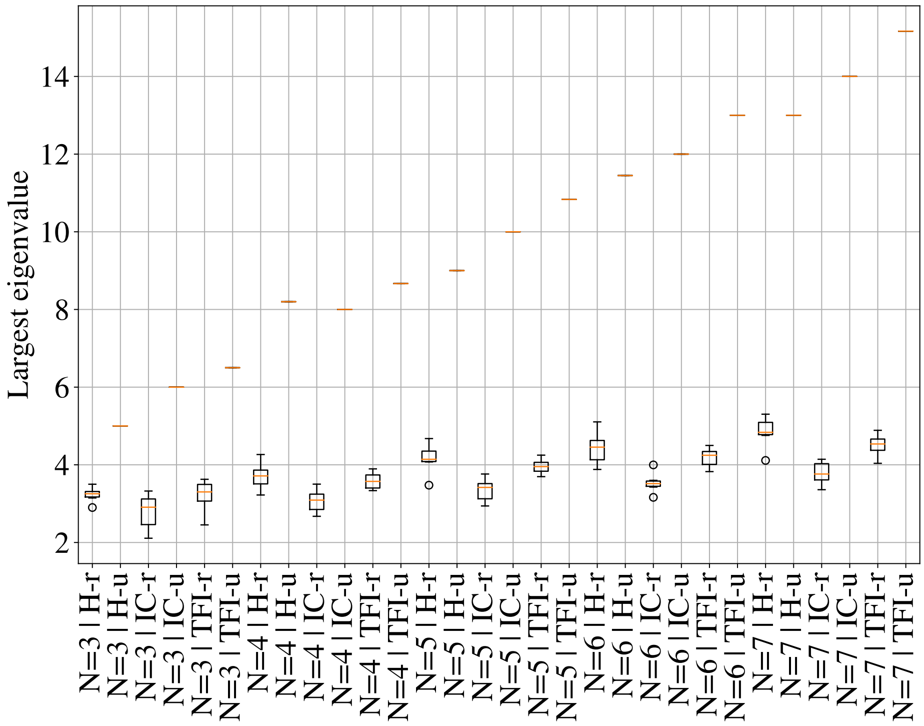

Figure S4. Largest eigenvalue for the models studied in the paper over five seeds. Here, H, IC and TFI refers to the Heisenberg, Ising Chain and Transvers-field Ising chain models, respectively. The "-u" and "-r" suffix refers to if the coefficients are uniform (equal to one) or randomly drawn from a standard normal distribution.
